# Editorial: DNA repair and immune response

**DOI:** 10.3389/fimmu.2022.1034689

**Published:** 2022-09-30

**Authors:** Paulo José Basso, Clarissa Ribeiro Reily Rocha, Erik A. L. Biessen, Ingrid Van der Pluijm, Carlos Frederico Martins Menck, Niels Olsen Saraiva Câmara

**Affiliations:** ^1^ Laboratory of Immunobiology of Transplantation, Department of Immunology, Institute of Biomedical Sciences, Universidade de São Paulo, São Paulo, Brazil; ^2^ Department of Clinical and Experimental Oncology, Federal University of Sao Paulo (UNIFESP), São Paulo, Brazil; ^3^ Department of Pathology, Cardiovascular Research Institute Maastricht (CARIM), Maastricht, Netherlands; ^4^ Department of Molecular Genetics and Department of Vascular Surgery, Erasmus University Medical Center, Rotterdam, Netherlands; ^5^ Department of Microbiology, Institute of Biomedical Sciences, Universidade de São Paulo, São Paulo, Brazil; ^6^ Laboratory of Clinical and Experimental Immunology, Division of Nephrology, Universidade Federal de São Paulo, São Paulo, Brazil

**Keywords:** DNA damage response, DNA lesions, inflammation, immune cells, cancer

## 1 Introduction

### 1.1 Duality of the relationship between DNA damage responses and immunity: Health and disease

Genetic stability allows for the reliable transfer of genetic information to succeeding generations. In this regard, a complex and overlapping protein network operates to fix the DNA damage caused by internal or external stressors (1). This so-called “DNA Damage Response” (DDR) is a fine-tuning process and works actively to guarantee systemic homeostasis.

The imbalance between DNA damage and repair mechanisms accelerates the aging process and increases the risk of developing several age-related diseases such as cancer, cardiovascular diseases, and neurodegeneration. However, not all genetic modifications are harmful and some are essential for the correct functioning of the organism. Somatic mutations, for instance, guarantee the diversification and broad repertoire of immune receptors, ensuring an effective protective immunity against a wide variety of pathogens. Thus, the cooperation between DDR and the immune system has been discussed and offers a new field of investigation in which in-depth comprehension may provide new insights into the cellular and molecular mechanisms of inflammatory diseases.

#### 1.1.1 An effective cooperation between DDR and immunity promotes health

**Figure 1:**
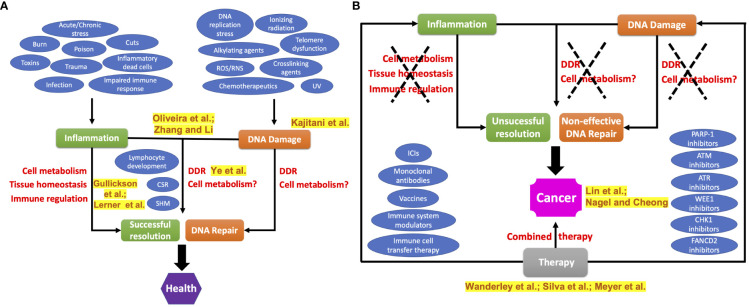
Relationship between inflammatory response and DNA damage in health and disease. **(A)** DNA damage response and immunity are closely related systems. They work together to deal with several negative stressors as well as to perform some physiological processes that require deliberate DNA breaks and rearrangement (e.g., lymphocyte receptor assembling, CSR, SHM), yielding a diverse set of immune receptors and antibodies capable of recognizing a broad range of antigens. **(B)** Chronic low-grade inflammation induces persistent DNA damage, and vice-versa, leading to cancer development. Current cancer treatment options envisage approaches targeting inflammatory or DNA-damaging agents separately. However, combined therapies have gained special attention as a potential strategy to improve the efficacy of cancer treatment. Here, we positioned the respective study from the manuscript collection closely to its main topic with the names of the authors highlighted in yellow, featuring the importance of the study’s contribution to the field. CSR, class switch recombination; DDR, DNA damage response; ICIs, immune checkpoint inhibitors; SHM, somatic hypermutation; UV, ultraviolet (radiation).

When DDR is unable to deal with extended and irreparable DNA lesions, cellular alterations (e.g., misplaced cytosolic DNA fragments) will be promptly recognized by innate immune receptors and initiate or amplify inflammatory responses that will work to remove potentially malignant cells and, thus, preventing the perpetuation of DNA damages **(** **Figure 1A** **)**.

Although there are DNA damage sensors that elicit an immune response (e.g., Ku70, DNA-dependent protein kinase, MRE11, Rad50, RNA polymerase III, and DExD/H-box helicase 41), there are also effector components that play multiple cellular roles, including in DDR and inflammation such as the poly(ADP-ribose) polymerase-1 (PARP-1), the enzyme mutY Homolog (MUTYH), and the 8-Oxoguanine DNA glycosylase-1 (OGG1) (2). In this edition, Oliveira et al. also discuss the role of apurinic/apyrimidinic endonuclease 1/redox effector factor 1 (APE1/Ref-1), a member of the base excision repair (BER) pathway, as another regulator of immunity through control of cellular signaling, redox status, senescence, and chromatin demethylation. Furthermore, Zhang and Li report on relevant observations regarding the structure and function of a versatile protein family belonging to the E3 ubiquitin ligase superfamily, called Pellino (Pellino-1, Pellino-2, and Pellino-3). The authors dissect Pellino’s roles in the pattern recognition receptor, tumor, and microRNA signaling pathways. Ye et al., in turn, notably discuss current evidence on how DDR components communicate with both innate and adaptive immunity.

The adaptive arm of the immune system, composed essentially of lymphocytes and their subsets, requires random and purposeful DNA breaks to generate a vast repertoire of receptors that will recognize a broad range of antigens from infectious agents. Even after the receptors have been correctly produced during lymphocyte development, the DNA breaks may continue later in the lymphocyte’s life. These processes are called class switch DNA recombination (CSR) and somatic hypermutation (SHM) and are essential for the generation of immunological memory and the production of highly specific antibodies. Any impairment of these mechanisms leads to critical DDR deficiency-driven immune system disorders and these are examined here by Gullickson et al.

CSR is a molecular mechanism that allows changing of antibody class from one to another (e.g., IgM to IgG or IgA). Previous studies showed that CSR requires DNA mismatch repair (MMR) and non-homologous end joining (NHEJ) pathways to replace the constant regions of immunoglobulins (Jhamnani et al.). On the other hand, SHM is a mechanism that introduces new mutations into antibody regions that recognize the antigens to increase antibody affinity (Pilzecker and Jacobs). This process is mediated and dependent on activation-induced cytidine deaminase (AID), which putatively distributes the mutations at G/C and A/T bases in similar ratios. However, even with the development of high throughput sequencing technologies, studying both CSR and SHM mechanisms remains challenging considering the limitations of current *in vitro* and *in vivo* approaches. Here, Lerner et al. describe that the Ramos cell line, a commonly G/C mutation-prone *in vitro* model used to evaluate SHM mechanisms, is capable of recapitulating the mutations at A/T bases by inhibiting ubiquitin-specific protease 1 (USP1) deubiquitinase activity and reestablishing the balance between proliferating cell nuclear antigen (PCNA) ubiquitination and deubiquitination.

Dissecting physiological and pathophysiological mechanisms is still the main path to the development of new therapeutic strategies against cancer and other illnesses. Here in this issue, Kajitani et al. show that the transgenic expression of cyclobutane pyrimidine dimers (CPDs) or 6-4 pyrimidine-pyrimidone photoproducts (6-4PPs) photolyases in nucleotide excision repair (NER)-deficient mice exposed to UVB completely abrogated or reduced the inflammation, epidermal thickness, and cell proliferation in basal keratinocytes, indicating a central role of these cells in the control of responses to UVB-induced DNA lesions.

#### 1.1.2 When the cooperation between DDR and immunity fails: the disease

When the organism is unable to counteract the high number of DNA damage through DDR and immune responses, a chronic low-grade inflammatory environment is established. This process is considered one of the strongest risk factors for cancer development **(** **Figure 1B** **)**. In this issue, Cheong and Nagel not only review the cancer risk from dysfunctional DDR and immunity, but also discuss the influence of other factors such as genetics, aging, environment, lifestyle, circadian rhythm, and diet. The authors also emphasize the role of ongoing technologies in the advancement of knowledge in the DDR-immunity axis. In fact, the use of technology to determine both DDR and immune profiles is useful to improve clinical management since the intra- and inter-tumor heterogeneity among the patients remains a challenging concern. For this purpose, Lin et al. find two different profiles of patients with hepatocellular carcinoma based on their genomic landscape of DDR. The so-called “DDR-activated group” was categorized by patients with aggressive cancer and poor outcomes, while the “DDR-suppressed group” had a better prognosis.

Immune checkpoint inhibitors (ICIs) have revolutionized cancer therapy with their capacity for modulating the immune response (3). Because their use as single agents has shown unprecedented clinical benefits, the present state-of-the-art approach has focused on combining them with different anti-tumoral drugs to improve clinical outcomes. Wanderley et al. bring to light the potential of using ICIs with PARP1 inhibitors. As previously mentioned, PARP1 has multiple cellular functions, acting as a DDR agent and immune cell modulator. A limitation of ICIs relies on their effectiveness in less immunogenic tumors. In this regard, Silva et al. point out that ICIs also show better prognosis when used in MMR-deficient tumors and discuss ongoing approaches to increase ICI sensitivity in homologous recombination (HR)-deficient tumors. On the other hand, increased HR rates can also mitigate the immune response to cancer. Meyer et al. observe that ALDH1-positive breast cancer stem cells in phase S are resistant to radiation by increased HR activity, being a potential target to increase sensitization to radiotherapy.

## 2 Conclusion and perspectives

As evidenced by the latest cutting-edge research, DDR-related proteins are not only restricted to DNA repair processes, but also participate in other cellular circuits that regulate immune cell signaling and function. The crosstalk between DDR and immune response has only begun being dissected, opening new perspectives for understanding regulatory mechanisms controlling inflammation and providing new potential strategies to treat inflammatory (age-related) diseases by targeting the DDR-immunity axis.

## Author contributions

PB wrote and drafted the whole editorial. CR, EB, IP, CM, and NC reviewed and approved the editorial.

## Funding

All our studies are supported by the Fundação de Amparo à Pesquisa do Estado de São Paulo (FAPESP 2017/02564-7; 2019/19435-3; 2021/03182-9); The role of DNA damage and mitochondrial function in vascular, immune and neurological aging within the ‘NWO-FAPESP joint Call for Proposals Healthy Ageing’, project number 4ST0O2OO1, Conselho Nacional de Desenvolvimento Científico e Tecnológico (CNPq), Coordenação de Aperfeiçoamento de Pessoal de Nível Superior (CAPES), financial code 001, CAPES-COFECUB international collaboration program.

## Conflict of interest

The authors declare that the research was conducted in the absence of any commercial or financial relationships that could be construed as a potential conflict of interest.

## Publisher’s note

All claims expressed in this article are solely those of the authors and do not necessarily represent those of their affiliated organizations, or those of the publisher, the editors and the reviewers. Any product that may be evaluated in this article, or claim that may be made by its manufacturer, is not guaranteed or endorsed by the publisher.
